# Parliament and Publications

**Published:** 1911-09-30

**Authors:** 


### PARLIAMENT AND PUBLICATIONS.

### FINAL REPORT OF THE ROYAL COMMISSION ON TUBERCULOSIS.

A final report on the investigation of viruses obtained from cases of human tuberculosis has been issued by the Royal Commission on Tuberculosis. The report gives the results of the investigation of tubercle bacilli from fifty-four cases of human tuberculosis. In two instances human and bovine tubercle bacilli were isolated from the same patient, and these were in each instance associated together in the same lesion. The cultures isolated from the other cases fall readily into one or other of two groups, one containing bacilli indistinguishable from bovine tubercle bacilli, the other bacilli (human tubercle bacilli) which grow more luxuriantly and have lower virulence for the rabbit and much lower virulence for the calf than the bovine tubercle bacillus. Bovine tubercle bacilli were isolated from four out of ten cases of alimentary tuberculosis in children and from two cases of pulmonary tuberculosis (sputum). Human tubercle bacilli were isolated from the remaining forty-six cases, *i.e.* six cases of alimentary tuberculosis, thirty-one of pulmonary tuberculosis, and nine of various other forms of tuberculosis. The milk from four nursing women regarded as suffering from pulmonary tuberculosis did not produce tuberculosis in guinea-pigs; the diagnosis of pulmonary tuberculosis was confirmed in one case only by the demonstration of tubercle bacilli in the sputum by guinea-pig inoculation.—Cd. 5790, price 5s. 3d.

### ASYLUM NURSES AND THEIR HOURS OF DUTY.

The Select Committee to which the Asylums Officers (Employment, Pensions and Superannuation) Bill was referred has issued a report. The Committee is of opinion that in a number of cases the hours of attendants and nurses in asylums are excessive and ought to be diminished. Eighty hours and more of such work in a week cannot be defended, but the Committee thinks the statutory reduction to sixty hours a week in all asylums is in most cases too great a departure from existing customs, and does not at present appear practicable. In the view of the Committee an average of seventy hours a week for the day staff and sixty hours for the night staff should not be exceeded. These figures approximate to those recommended by the Lunacy Commission.

### OSBORNE.

The report by the House Governor and Medical Superintendent at Osborne for the year ending on March 31, 1911, shows that the total admissions of ladies and officers and the average daily number in residence were below last year's figures. The decrease is accounted for by (1) insufficient knowledge of the benefits of Osborne among sick officers; (2) the very large reduction in the invaliding home of officers and soldiers which has taken place during the past three or four years; (3) the establishment of new homes and institutions for sick officers in the hills in India, where many cases pass their period of convalescence instead of coming home.

